# History of national sex surveys in Europe

**DOI:** 10.12688/openreseurope.16574.1

**Published:** 2023-11-20

**Authors:** Osmo Kontula, Soazig Clifton

**Affiliations:** 1 Population Research Institute, Family Federation of Finland, Helsinki, FI-0010, Finland; 2 School of Health and Wellbeing, University of Glasgow, Glasgow, UK

**Keywords:** Sex Surveys, History, Europe, National Surveys

## Abstract

The historical background of European national sex surveys dates back to the late 1960s and early 1970s when the first such surveys were conducted in Europe. That was an era of Western sexual revolution, a time of increasing openness to discussion and debate of sexual issues, and legal reforms in many countries. During this period, there was a growing interest in understanding sexual behaviour, attitudes, and modern contraceptive use in the general population. Evidence was also needed for sexuality education that was emerging in some European countries. Despite this need for evidence, only a few countries (Sweden, Finland, France) conducted national surveys on the topic in the early 1970’s, becoming pioneers in national sex surveys in Europe and in the world. It was the HIV and AIDS epidemic of the 1980s that gave more researchers and funders the impetus to conduct sex studies in many European countries. As a result, medical perspectives on sexuality became prioritised around Europe. Balancing the perspectives of the diverse disciplines (including public health, sociology, psychology) that use the data from national sex surveys remains a challenge for the modern surveys. The European Sexual Medicine Network (ESMN) is a part of European cooperation in science and technology (COST), which includes a strand of work on national sex surveys among the adult population. Since 2021, a subgroup of nationally representative sex surveys, co-chaired by Osmo Kontula and Hanneke de Graaf have worked together to further the field. This submission is based on a review of the literature, combined with Kontula’s personal reflections following a history of participation in European collaboration in this field since the early 1990s.

## Introduction

The first European national sex surveys were conducted in the late 1960s and early 1970s, during an era of Western sexual revolution that raised public discussion and debate of sexual issues and brought about legal reforms in many countries. There was increasing interest in evidence on sexual behaviour, attitudes and modern contraceptive use in the general population ^1^. Evidence was also needed for sexuality education which was being introduced in some European countries ^2^. Despite the clear need for research data, only three European countries (Sweden ^3^, Finland ^2^, and France ^4^) conducted national surveys at the time, becoming pioneers in the field of high-quality representative sex surveys, both in Europe and internationally. They were influenced by Kinsey's study but they used different methods and made no comparisons to Kinsey's results.

## European sex surveys in the late 1960s and early 1970s

The first sex survey in Sweden ^3^ (‘ *On sexual life in Sweden: Values, norms, behaviors in sociological interpretation.' | 'Om sexuallivet i Sverige: Värderingar, normer, beteenden i sociologisk tolkning'*) was conducted in 1969, led by Hans L. Zetterberg and published by the Swedish Institute for Opinion Polls (SIFO). The survey investigated sexual and marital issues, and was the first sex survey in the World based on a real representative national random sample.

The Finnish sex survey ^2^ (later FINSEX) was conducted in 1971, led by Kai Sievers, Osmo Koskelainen and Kimmo Leppo. The study focused on heterosexual sex within the context of marriage (and extramarital sex) and the questionnaire included sexual activity and disorders, youth sexuality experiences and masturbation, birth control and contraceptive methods, and information and attitudes related to sex, with a sub-study on fertility and family planning. The study involved personal interviews with self-administered questionnaires, which had different versions for women and men. The sample (N=2200) in the age range of 18 - 54 was drawn randomly from the central register of the population. The interviews in the study were conducted by 1,000 midwives and health nurses. The response rate of 91.4% remains a “world record” for national sex surveys.

Pierre Simon’s Report of the first national sex survey in France (1972) ^4^ proposed a naturalistic approach to sexual behavior, enriched by considering sociological and social-psychological factors. The survey was conducted in the early 1970s, using quota sampling (N = 2,625). Like the Swedish and Finnish surveys, the Simon Report focused on heterosexual sex but not only to married couples. Questions on sexual relations were posed in terms of a sexual partner, either husband/wife or "boyfriend/ girlfriend." The psychological dimensions of these relationships were explored in more detail than the sexual acts performed. Simon strongly emphasizes the dimension of love, the couple, and the control of procreation. The questionnaire was based on a perspective of sexual health that assesses knowledge of contraception and biological mechanisms of sexuality. There were series of questions on attitudes toward contraception and abortion, on opinions and attitudes toward sexuality. The study was situated in the context of the introduction of oral contraception.

## Sex surveys in the 1980s and early 1990s

It was not until the late 1980s that these pioneering sex studies were followed up in Europe.

In the 1980s, after AIDS appeared, many European countries realized that, outside specific studies on gay men and injecting drug users, little was known about the prevalence of various types of HIV/AIDS risk-related behaviour, or sexual behaviour in general, in their national populations. There was a need to fill the information gap by conducting large-scale population surveys for the first time. In a sense, HIV/AIDS prevention and epidemiology legitimized research on sexuality, which was somewhat devalued and under-developed in the past.

The European surveys completed in the context of HIV/AIDS between 1989 and 1992, with a focus on epidemiology and health education relating to sexually transmitted infections (STIs) and HIV/AIDS ^5^.

The biomedical perspective became dominant in sex surveys at this time ^6^, with the notable exception of the Finnish survey carried out in 1992, which belonged to a tradition of the human sexuality studies in the late nineteenth century conducted in the Nordic countries and the USA ^7^. The funding of the Finnish survey in the 1990s by the Academy of Finland, jointly by Social and Medical Committees, can be seen as representing generational perspective and a new tradition of sexual health in the country. In contrast to the early studies of the 1970s, the studies in the late 1980s and early 1990s moved away from considering sex purely in terms of heterosexual, marital sex. In the context of AIDS, public health concerns about gay men and anal sex reshaped the understanding of sexual activity, as described in detail by Michaels and Giami ^8^. The notion of a "type of partner" that allows for the possibilities of multiple partners and/or partners of either or both genders took precedence over a particular social relationship, marriage, which became one among multiple types of social relationships (married, cohabiting, regular, occasional, and casual). Epidemiological concerns highlighted the distinction between having one or multiple partners, replacing the moral opposition between marital and extramarital sexual activity. The need for precise epidemiological data on sexual contacts involving potential risk of infection led to the formulation of more explicit questions concerning a variety of sexual acts.

## European sex surveys in the early 1990s:

- Athens-KABP 1989, Athens-PR 1990, Belgium 1993, Finland 1992, France-ACSF 1992, France-KABP 1992, Germany (East) 1990, Germany (West) 1990, Germany (East & West) 1993, Great Britain 1991, Netherlands 1989, Norway 1992, Portugal 1991, Scotland 1992, Spain 1990, Switzerland 1992.

Among the most renowned and the largest scale nationally representative European surveys in the 1990s were the British (Natsal) and French (ACSF) surveys, both had around 20,000 respondents. The primary objectives of the British survey were directly related to developing public health responses to AIDS: (1) to help elucidate the transmission patterns of STDs and HIV; (2) aid in the "selection of appropriate and effective health education strategies for epidemic control". Despite the unexpected withdrawal of government funding, the survey went ahead (fielded 1990-1991) with funding from the medical research charity, the Wellcome Trust, who have funded the study ever since ^9^. Results were published in 1994 ^10, 11^, covering the extensive methodological development work, the rigorous research process, and the key findings.

The national survey of sexual behavior in France (ACSF) was fielded in 1991-92 ^8^. The survey was requested by the National Agency for AIDS Research to inform prevention policies due to HIV epidemic. The main objectives were: “Collecting basic information on the sexual behavior of the population, with the aims of devising more effective strategies for preventing AIDS in particular and sexually transmitted diseases in general and developing models for predicting the spread of the epidemic”. This same agency has continued its support in funding later French sex surveys ^12^.

The most important additions to the FINSEX survey in 1992 ^13^, in comparison with its 1972 predecessor, reflect the long-term trend toward "sexual optimism" increasingly considering sex as a positive and fulfilling experience that has characterized modern sex research from Havelock Ellis to Masters and Johnson ^8^. In Sweden, the former Public Health Institute of Sweden undertook, on assignment from the government, the study “Sex in Sweden” in 1996. In Czech Republic the study sexual Behaviour of Czechs was funded in 1993 directly by Ministry of Health from the AIDFS Fund. Later sex surveys in Czech were financed by pharmaceutical firms (Pfizer and Chemie). In Norway, the studies were financed by the Norwegian Directorate of Health.

## European concerted action on sexual behaviour and risks of HIV infection in the 1990s

The World Health Organization (WHO) provided some European researchers in Athens, Portugal and West Germany an opportunity to meet in the early stages of this field of HIV/AIDS research (1987-1989) and to exchange ideas and contribute to the design of a survey protocol that was used in several developing countries ^5^. In the meeting in Portugal in November 1991 an agreement was reached among the 35 participants from 12 countries that an European Concerted Action on Sexual Behaviour and Risks of HIV Infection would undertake two separate activities:

1) An investigation of the conceptual perspectives from a European viewpoint; and
2) A comparison of empirical data about sexual behaviour and the risks of HIV from general population studies throughout Europe.

## An important European book of conceptual perspectives

An outcome of the first activity was a number of expert meetings and a book published in 1997, “Sexual Interactions and HIV Risk: New Conceptual Perspectives in European Research” ^14^. This work, based on the findings from work coordinated by the Centre d'Etudes Sociologiques in Brussels, offered a social critique of the theories and perspectives which had been brought to bear in the study of sexual risk behaviour and HIV. Leading European researchers offered a conceptual framework for analysis based on sexual interactions and their social context. Authors of that book were researchers from eight European countries with different backgrounds and theoretical references. One key message was that for more successful intervention strategies, it is necessary to move from individual to social and interaction-oriented perspectives. This new perspective had an impact on the content and themes of the European sex surveys of those times.

## Comparison of empirical data about sexual behaviour and the risks of HIV from general population studies throughout Europe

The second action, funded through the European Union (EU) Biomedical and Health Research Programme (BIOMED), brought together data from eleven Countries (sixteen surveys) in cross-national analyses that were eventually published as a collaborative effort book in 1998 ^15^. The objectives, tasks and working procedures for the project were determined by the Centre d’études sociologiques (CES) of the Brussels-based Facultés universitaires Saint-Louis which coordinated the project, aided by a steering committee, with collaboration from investigators from the national surveys. It was the first time that such systematic comparisons was undertaken in Europe. As with any post-hoc harmonisation effort, the study was constrained by the fact that the surveys had been designed independently before the Concerted Action had started and were implemented according to distinct research protocols, given each team's specific aims and funding and time constraints. Because of this, there was no possibility to improve the comparability of the survey questionnaires and protocols. Given the urgent need for data to contribute to the HIV/AIDS response, researchers and funding bodies could not spend much time harmonizing their approaches with other countries ^15^. Many surveys included in this comparative research were not sex surveys as such but primarily health studies. The Dutch (1989), British (1991), Finnish (1992) and French ACSF (1992) surveys were the only European surveys that were more straightforwardly in the tradition of sex research.

This comparative analysis aimed to present key data on sexual behaviour and attitudes towards HIV/AIDS from 16 population surveys carried out in 11 European countries between 1989 and 1993. These counties were Greece (Athens), Belgium, Finland, France, Germany, Great Britain, Netherlands, Norway, Portugal, Scotland, Spain and Switzerland. Scientific information about the prevalence of different kinds of sexual behaviour was analyzed in ways relevant to a better understanding of the epidemic and its dynamics.

To fulfil the objectives of drawing empirical, methodological and theoretical conclusions from these disparate experiences, different themes were defined, and specialists of these themes were asked to design key research questions that could be studied cross-nationally. A computerized data base (the European Bank of Indicators of Sexual Behaviour and Attitudes towards HIV/AIDS Risk – EBI-AIDS) was set up to select relevant indicators and questions from the various surveys that were to be compared. Thirty common variables were defined, and each survey investigator was asked to create them in their data file to describe the samples and populations investigated and explain the dependent variables under study.

## Second European collaboration: The new encounter module (NEM) surveys in the 1990s

The European collaboration in national sex surveys in the beginning of the 1990s was followed with another collaborative project the “New Encounter Module for following-up HIV/AIDS prevention in the general population surveys”, also called the “New Encounter Module project” or “NEM project” ^1^. This was funded by the EU “EU against AIDS” programme and coordinated by Michel Hubert in Brussels.

NEM project aimed to further a cross-national perspective on the HIV/AIDS epidemic and prevention in Europe by designing a common survey protocol (not published) for use in the next surveys on sexual behaviour and HIV/AIDS prevention carried out in Europe ^1^. The participants in this project were the research teams in charge of the next general population surveys on sexual behaviour and HIV/ADS prevention to be carried out in Europe. NEM project organized several European meetings in Brussels 1994-1997. The project decided to adopt a relationship-based approach that consisted in asking specific questions about the last new encounter in which the respondents were involved. This approach had the advantage of providing precise information about the state of the population's response to HIV risk in the most recent relationships, as well as about the potential spread of HIV infection. It included key indicators of sexual behaviour and HIV/AIDS prevention. Project members worked to promote the use of the common set of indicators and survey questions, particularly the New Encounter Module (NEM), in as many countries as possible. The complete module and protocol were used in ten surveys in nine countries 1997 - 2001 ^1^: Norway, England, Germany, France, Switzerland, Portugal, Spain, Italy, and Greece. Finland and Slovenia also carried out surveys but not including all items in the frame of the NEM project. Later also Georgia joined the NEM project. The target group was the 18 – 49 years. The national data was standardized so that they could be used on a comparable base and data analyses could be carried out. In addition to national reports NEM surveys were used for European comparisons in several publications. The Council of Europe published two reports of sexual behaviour among young Europeans that included data from NEM survey **s** ^16, 17^ alongside survey results from Denmark, Iceland, Sweden, St. Petersbourg, Finland and Estonia. Other outputs using the NEM project data were “Bi- and Homosexuality in the National Surveys in Europe” using the national survey data from 13 (mostly NEM) countries ^18^, and “Between Sexual Desire and Reality: The Evolution of Sex in Finland”, which included comparisons to eight European countries.

## Other initiatives in the 1990s

In some European countries, including Latvia, the International Conference on Population and Development in 1994 in Cairo gave a boost to plan other national sex surveys, with surveys financially supported by United Nations organizations as well as the International Planned Parenthood Federation (IPPF). Eastern Europe countries were selected among the countries that needed international research funding. One important aim of these surveys was to promote female sexual and reproductive rights internationally. In Latvia, surveys were conducted on improving the low birth rate and demographic situation.

There was a separate collaboration and comparison of national sex survey between four Nordic countries, published in “Sexual Trends in the Baltic Sea Area” ^13^. It included sex surveys in four cultural areas in the Baltic region: Two Nordic countries (Finland (1971, 1992, and 1999), Sweden 1996 (Levin et al., 1998)), and two that represented former Soviet Union areas (St-Petersbourg in Russia (1996), and Estonia (2000)). Each of the four areas is geographically adjacent to the Baltic Sea, and the histories are intertwined in many ways. Each of the four areas has its own language, and the ethnic background and culture of each region’s population is different. This comparison illustrated the impact of economic, political, and cultural factors on sexual lifestyle. Of all factors that were studied, six indicated that the change in sexual values and habits occurred first in Sweden and proceeded from there to Finland, next Estonia and then St. Petersburg. These trends were openness about sex in the childhood home, sexual initiation at a young age, acceptance of sex without love, lifetime polygamy among women, low number of parallel relations, and masturbation.

## New sex research trends in the 2000s

During the 1990s, new public health concerns and social movements led to a dramatic reformulation of conceptions of both heterosexuality and homosexuality. The review by Michaels and Giami ^8^ demonstrated that there was no longer any assumed link between sexual acts and sexual relationships in sex surveys, a change which was carried forward into the 2000s. The link between sexual practices and sexual relationships evolves with changes in society, and sex surveys reflect these changes as they attempt to measure them. The textbook “New Views on Sexual Health: The Case of Finland” gave an important inspiration to continue FINSEX surveys in 2007 and 2015 ^19^, funded by the Ministry of Social Affairs and Health. The key aim of these surveys was to study the determinants of sexual wellbeing, not risky sexual behaviour.

In Spain in the 2000s the aim of the new National Strategy for Sexual and Reproductive Health was to improve the sexual health status of the general population ^20^. There was a need for the agreement on the definitions of sexual health and sexual rights. It has had an impact on key issues and selected measures in sex surveys in Spain ^12^. In France, the social context of sexuality became an important research theme in the 2000s ^21^. A new trend in some European countries, including Norway, has been to study issues that are problems in perspective of sexual rights, including a focus on sexual violence (especially following the Me Too movement which gained widespread recognition in 2017), unwanted pregnancy, and sexual rights for suppressed and stigmatized groups including sexual minority. In the Netherlands, the topics of sexual harassment and violence are gaining attention. Later exploring reasons for the decline in sex, imbalance in sexual desire, and issues of consent have become more prevalent in European sex surveys ^12^.

In Sweden, the survey in 2017 covered sexual and reproductive health and rights and focused on sexual and reproductive health as health determinants from a perspective of public health, equity, and gender equality. In Czech population’s paraphilic preferences were measured in 2016. This study stimulated the development of new health services and policy for people with paraphilic interests and problematic sexual behaviour in the Czech Republic ^12^. In European sex surveys the current methodological challenge is to recruit samples that are representative of the general population, and that also represent the diversity of sexualities and sexual experience.

## Recent and future collaborative work

Since the 1990s, there have been several attempts to start a new European collaboration among sex researchers, including proposals (not funded) to include a module on the European Social Survey in 2012 and 2014 and Rutgers meetings in 2015 and 2016. These working groups have finalized European survey instruments with number of key items ^12^, however lack of common funding for European data collection has been a block to including these instruments in a cross-national survey.

This collaboration was developed further, via the national sex surveys subgroup of the European Sexual Medicine Network (ESMN), funded by the European Cooperation in Science and Technology (COST). This group met in October 2021 in Prague, including representatives from ten European countries to share learning and explore collaborative opportunities (Germany, Latvia, Finland, Netherlands, Belgium, Norway, Czech Republic, Spain, Sweden, Britain, and France).

Beyond Europe, the World Health Organization has recently spearheaded efforts to develop an international brief sexual health instrument that could be included to collect basic sexual health data in surveys globally ^22, 23^. Discussions continue around whether this could be used for national comparisons in the ESMN sub-group participating European countries.

## Conclusion

Masturbation, marital sex, and homosexuality were the focus of the American Kinsey reports in the 1940s and 1950s. In Europe, we have seen striking trends in the conceptualization of sexual health as relevant to national sex surveys, from the early surveys in the late 1960s and 1970s which considered heterosexual marital sex and contraception, to the biomedical and risk-based studies HIV/AIDS era epidemiological studies in the 1980s and 1990s, then a focus on other sexually transmitted infections and reproductive health and later on in the 2000s to studies which have considered sexual health much more broadly to also include topics relating to sexual rights, sexual violence, harassment, and pleasure. However, there have been notable exceptions, for example in the Finnish study, which included pleasure and viewed sex as a positive, rather than risky, experience early on.

The sex survey community has a strong history of collaborative working in Europe; however, a lack of cross-national funding has blocked recent attempts to build on the comparative research projects of the 1990s, which would further our understand of the impact of context on sexual health in the 2020s. These efforts are ongoing, especially with the recent development of an international survey instrument by the World Health Organization.

There has been a significant change in the reporting of sexual studies since the 1990s. Instead of extensive books, the results have been published mainly as science articles. There are also more and more (diversity) scientific international journals that have some special interest in sexuality research and in sex surveys. The picture they form of each country's sexual culture and its changes remains fragmented.

## Ethics and consent

Ethical approval and consent were not required.

## Data Availability

No data are associated with this article.
