# Supplementary material for: History of national sex surveys in Europe
Source: Open Res Eur. 2023 Nov 20;3:199. [Version 1] doi: 10.12688/openreseurope.16574.1 (PMC11031641; doi:10.12688/openreseurope.16574.1)
Supplement: Peer review history

# Reviewer report: Reviewer response for version 1

**DOI:** 10.21956/openreseurope.17890.r36334

**Published:** 2024-04-19

**Authors:** Jaime Barrientos Delgado

**Affiliations:** 1 Universidad Alberto Hurtado, Santiago, Santiago Metropolitan Region, Chile

This is an interesting, necessary, and well-written paper on the history of sexuality surveys in Europe.

 To date, much of the information contained in this paper is in various places, out of order. Therefore, this paper is an important and necessary document. This paper describes this history, indicating the most important milestones, describing the characteristics of these milestones and how progress has been made from the 1970s until now. The authors describe this story in detail, alluding to facts available in various details.

 The authors are well acquainted with this history, and the surveys described. The authors make a history, but alluding at the same time to socio-cultural and theoretical issues. From my point of view and knowing an important part of this story, I believe that this is a paper that is very well written, and that in its current state does not require improvements. Therefore, and in my opinion, I would accept it for indexing in its current state.

Is the argument persuasive and supported by appropriate evidence?

Yes

Is the work clearly and cogently presented?

Yes

Is the topic of the essay discussed accurately in the context of the current literature?

Yes

Does the essay contribute to the cultural, historical, social understanding of the field?

Yes

Reviewer Expertise:

Sexology; Sex surveys; Social Psychology.

I confirm that I have read this submission and believe that I have an appropriate level of expertise to confirm that it is of an acceptable scientific standard.

# Reviewer report: Reviewer response for version 1

**DOI:** 10.21956/openreseurope.17890.r37670

**Published:** 2024-04-19

**Authors:** Richard Krueger

**Affiliations:** 1 Columbia University, New York, New York, USA

This is an important article describing the evolution of national sex surveys in Europe, ending with a pitch to such surveys being done by the World Health Organization.

 It is well organized, well written, with appropriate references.

 I would suggest that on page 4, 2nd to last paragraph on the right, that references be added for the Dutch, British, Finnish and French surveys.  

 As it stands the article is fine, but I am sure that the authors would agree that a broad expansion of such surveys to include other countries and areas in the world would be desirable although complicated to effectuate and the authors might make a stronger suggestion for this

Is the argument persuasive and supported by appropriate evidence?

Yes

Is the work clearly and cogently presented?

Yes

Is the topic of the essay discussed accurately in the context of the current literature?

Yes

Does the essay contribute to the cultural, historical, social understanding of the field?

Yes

Reviewer Expertise:

I have been involved with revising the ICD-10 and developing ICD-11 diagnostic criteria for sexual disorders and helped with several studies investigating the reliability of proposed criteria in many countries associated with the WHO.

I confirm that I have read this submission and believe that I have an appropriate level of expertise to confirm that it is of an acceptable scientific standard.

# Reviewer report: Reviewer response for version 1

**DOI:** 10.21956/openreseurope.17890.r37080

**Published:** 2024-02-26

**Authors:** Emma Slaymaker

**Affiliations:** 1 London School of Hygiene and Tropical Medicine, London, UK

This paper describes, in chronological order, the sex surveys that have been conducted in European countries since the 1960s. In terms of changes over time, I thought that three themes emerged: the geographical coverage of the surveys, the content of the surveys and the motivation and source of funding for these surveys. Key European collaborations are also described.

 It would have been interesting to see these themes discussed more explicitly - for all the surveys in each decade a review of the geographical coverage, the content and the motivations. The way the paper was structured perhaps prevented a fuller exploration and I did feel it would have been easier to follow if the collaborations had been discussed separately, after the review in chronological order. It would also be really useful to have a list of all the surveys mentioned in the paper, it would help follow the text, and would be a useful resource.

 Whilst this is a history of European sex surveys, the only comparison made with surveys elsewhere in the world is with the Kinsey study in the US but there are other points of comparison with more recent surveys elsewhere in the world that show the difference in the European approach. As well as the existence of cross-national European collaborations, I think it is worth mentioning the comparative wealth of information in Europe (as a result of the surveys discussed in this paper) when compared with other regions of the world. Related to that, it would also be good to summarise which countries have had regular surveys, and which have had none, or fewer surveys. There is also another reference to Kinsey in the conclusion but I am not sure what the point of comparison is there.

 The authors mention a literature review but no details of this have been given. Relatedly, no inclusion/exclusion criteria for surveys have been given and so it is not clear which surveys are counted. For instance, some of the Reproductive Health Surveys conducted in Eastern Europe and former Soviet republics during the 1990s contain some information on sexual behaviour of young people but were not considered here. I think the paper needs this information.

 A few specific points:

**Introduction:**

- Kinsey’s study is referred to but not referenced and it should be stated that this was a US study done some time before the European surveys being discussed.
- It would be useful to have a brief summary of what was known about sex in Europe, and what information sources existed prior to the surveys. 

**European sex surveys in the early 1990s:**

- This section starts with a list of the surveys which is  useful but not given elsewhere. The Czech survey is mentioned in the text but not listed whilst some of those listed are not described in the text.
- In the last para  - 'Czech' should be the 'Czech Republic'.

**Sex surveys in the 1980s and early 1990s:**

- This paragraph is missing something:
  "The European surveys completed in the context of HIV/AIDS between 1989 and 1992, with a focus on epidemiology and health education relating to sexually transmitted infections (STIs) and HIV/AIDS."

Is the argument persuasive and supported by appropriate evidence?

Partly

Is the work clearly and cogently presented?

Partly

Is the topic of the essay discussed accurately in the context of the current literature?

Yes

Does the essay contribute to the cultural, historical, social understanding of the field?

Yes

Reviewer Expertise:

epidemiology, demography, surveys, sexual behaviour, HIV, data harmonisation and secondary data analysis.

I confirm that I have read this submission and believe that I have an appropriate level of expertise to confirm that it is of an acceptable scientific standard, however I have significant reservations, as outlined above.
